# Hyperoxia promotes bronchopulmonary dysplasia via Noggin-mediated BMP4 antagonism and cellular senescence

**DOI:** 10.3389/fphys.2026.1761135

**Published:** 2026-04-20

**Authors:** Jiaxin Zhang, Jia Quan, Yifan Luo, Zongli Zhang, Tao Li, Shibing Xi

**Affiliations:** 1 Department of Pediatrics, Taihe Hospital, Hubei University of Medicine, Shiyan, Hubei, China; 2 Pediatric Research Institute, Hubei University of Medicine, Shiyan, Hubei, China; 3 Department of Pediatric Intensive Care Unit, Maternal and Child Health Hospital of Hubei Province, Tongji Medical College, Huazhong University of Science and Technology, Wuhan, Hubei, China

**Keywords:** BMP4, bronchopulmonary dysplasia, cellular senescence, hyperoxia, Noggin

## Abstract

Bronchopulmonary dysplasia (BPD) represents the most prevalent chronic pulmonary complication in preterm infants, with incompletely understood pathophysiological mechanisms. Hyperoxia exposure constitutes a major risk factor for BPD development, inducing cellular senescence that impairs alveolar maturation. While senescence is predominantly mediated by the p53/p21 signaling pathway, upstream regulatory mechanisms remain inadequately defined. This study aimed to identify critical genes through bioinformatics and elucidate the molecular mechanisms by which the Noggin-BMP4 signaling axis mediates cellular senescence in BPD pathogenesis. Integrating BPD transcriptomic datasets with aging-related databases via WGCNA, Noggin was identified as a hub gene linking BPD and cellular senescence (AUC = 0.80). In HPMECs exposed to 85% hyperoxia, Noggin expression increased approximately 2.5-fold (mRNA) and 2.0-fold (protein), while BMP4 decreased to 50% of controls, accompanied by elevated p53 and p21 expression and positive SA-β-gal staining. Noggin silencing restored BMP4 expression and significantly attenuated hyperoxia-induced p53/p21 upregulation, suggesting that Noggin promotes senescence by suppressing BMP4. In a neonatal rat hyperoxia-induced BPD model, alveolar simplification was observed alongside a threefold increase in Noggin mRNA, a reduction of BMP4 to 30% of controls, and elevated p53/p21 at day 14, corroborating the *in vitro* findings. These findings suggest that hyperoxia upregulates Noggin to antagonize BMP4 signaling, thereby activating p53/p21-mediated senescence and contributing to alveolar developmental arrest. The Noggin–BMP4 axis may represent a potential therapeutic target for BPD.

## 1 Introduction

Bronchopulmonary dysplasia (BPD) represents the most prevalent chronic pulmonary complication affecting preterm neonates, characterized by impaired alveolar formation, disrupted pulmonary angiogenesis, and simplified lung architecture (Thébaud et al., 2019). Despite ongoing advancements in perinatal medical care, the incidence of moderate to severe BPD remains above 86% among extremely preterm infants born at 23–24 weeks of gestation (Geetha et al., 2021; Siffel et al., 2021). Affected infants endure significant long-term sequelae, including prolonged oxygen dependence, pulmonary hypertension, and recurrent respiratory infections, with respiratory dysfunction potentially persisting into adulthood (Davidson and Berkelhamer, 2017). However, due to an incomplete understanding of BPD’s underlying pathophysiology, effective clinical prevention and therapeutic strategies are currently lacking.

Exposure to elevated oxygen concentrations constitutes the primary risk factor for BPD development (Choi et al., 2021; Kimble et al., 2022). Fetuses adapted to the hypoxic intrauterine environment (approximately 4% O_2_) experience relative hyperoxia even at normal atmospheric oxygen levels (21% O_2_) (Obst et al., 2022). Hyperoxia induces excessive generation of reactive oxygen species (ROS), while the antioxidant defense mechanisms in preterm infants remain immature, resulting in oxidative stress injury (Lembo et al., 2021). This hyperoxic environment disrupts the regulatory balance of genes involved in pulmonary angiogenesis, provokes sustained inflammatory responses, and damages vascular endothelial and alveolar epithelial cells, thereby compromising the integrity of the pulmonary microvasculature (Stenmark and Abman, 2005). Notably, ROS can induce DNA damage and initiate specific signaling cascades that promote cellular senescence (Barnes et al., 2019).

Cellular senescence is defined as a state of irreversible cell cycle arrest predominantly governed by the p53-p21 and p16INK4a-Rb signaling pathways. Upon DNA damage or oxidative stress, the tumor suppressor p53 is activated and transcriptionally upregulates its downstream target gene CDKN1A, which encodes p21 (Westin et al., 2011; Mijit et al., 2020). The p21 protein inhibits cyclin-dependent kinase complexes, arresting cell cycle progression at the G1/S checkpoint and culminating in permanent cell cycle cessation (Yan et al., 2024). Senescent cells are characterized by enlarged size, flattened morphology, and elevated senescence-associated β-galactosidase (SA-β-gal) activity (Valieva et al., 2022). Recent investigations have implicated cellular senescence in the pathogenesis of chronic obstructive pulmonary disease and idiopathic pulmonary fibrosis (Schafer et al., 2017; Barnes et al., 2019). Emerging evidence confirms that hyperoxia induces senescence in pulmonary tissue cells (Koloko Ngassie et al., 2025); however, whether this effect specifically targets pulmonary microvascular endothelial cells and constitutes a critical factor impeding alveolar development remains to be elucidated. Crucially, the exact molecular mechanisms linking hyperoxia-induced endothelial senescence to disrupted alveolar development—particularly the potential involvement of the Noggin-BMP4 signaling axis—represent a significant research gap in BPD.

Bone morphogenetic protein 4 (BMP4), a pivotal member of the transforming growth factor-β (TGF-β) superfamily, plays an essential regulatory role in lung development (Calthorpe et al., 2023). BMP4 is abundantly expressed in the distal embryonic lung bronchial epithelium and mesenchyme, where it modulates progenitor cell differentiation, bronchial branching morphogenesis, and pulmonary angiogenesis (Hyatt et al., 2002). Appropriate BMP4 signaling levels are indispensable for normal lung development. For instance, BMP4 regulates the differentiation of alveolar type II (AT2) epithelial cells into alveolar type I (AT1) cells (Chung et al., 2018). Dysregulation of BMP4 signaling is closely associated with various pulmonary pathologies, including hypertension and fibrosis (Sun et al., 2008).

Noggin, encoded by the NOG gene, is a key secreted antagonist of BMP signaling. It binds BMP4 with high affinity, occluding its receptor-binding sites and thereby preventing interaction with cell surface receptors (Groppe et al., 2003). During early embryogenesis, Noggin plays a critical regulatory role in lung development by antagonizing BMP4 activity to induce lung branching morphogenesis (Weaver et al., 2000; Que et al., 2006). Nevertheless, prior studies have predominantly focused on Noggin’s direct effects on epithelial cell differentiation, with limited attention to its regulatory influence on pulmonary microvascular endothelial cell fate under oxidative stress conditions. Importantly, the Noggin-BMP4 signaling axis is highly context-dependent, and given that endothelial cells constitute a significant source of BMP4 (Wertheimer et al., 2018), alterations in their functional state may affect alveolar epithelial cell fate via paracrine mechanisms. This vascular-epithelial crosstalk remains to be elucidated in the context of BPD.

Although previous research has independently delineated the roles of hyperoxia, cellular senescence, and BMP signaling in BPD, the integrated regulatory mechanisms linking these factors remain unclear. Accordingly, the present study employs a multi-tiered experimental approach. We aim to elucidate the molecular mechanisms by which hyperoxia upregulates Noggin expression to antagonize BMP4 signaling. We hypothesize that this antagonism subsequently activates the p53-p21 pathway, inducing senescence in pulmonary microvascular endothelial cells. Furthermore, this investigation will explore how endothelial senescence disrupts angiocrine signaling, ultimately leading to impaired alveolar development in BPD. The findings are anticipated to provide novel theoretical insights into BPD pathogenesis from the perspective of vascular aging and to identify potential molecular targets for clinical intervention.

## 2 Materials and methods

### 2.1 Bioinformatics analysis

#### 2.1.1 Data acquisition and identification of differentially expressed genes

The GSE32472 dataset was retrieved from the GEO database (http://www.ncbi.nlm.nih.gov/geo), comprising peripheral blood transcriptomic data from preterm infants enrolled at a neonatal intensive care unit. Entry criteria included preterm birth below 32 weeks of gestational age and birthweight below 1,500 g (VLBW). BPD was diagnosed in 68 infants (40 mild, 13 moderate, and 15 severe), with 43 infants serving as non-BPD controls. Blood samples were collected at three time points: postnatal days 5, 14, and 28 (time points A, B, and C). Note that microarray data were not available for all patients at all time points, resulting in a total of 182 BPD samples and 112 control samples included in the differential expression analysis. Data pre-processing was performed using the GEOquery package, where we confirmed that the expression matrix had already been log2-transformed and normalized via the Robust Multi-array Average (RMA) method (range: 1.64 to 14.55). Given that all samples were processed within a single study and platform (GPL6244), batch effect correction was not required. DEGs were identified utilizing the limma package in R by fitting linear models and applying empirical Bayes moderation. To ensure a robust signal-to-noise ratio in this large cohort, thresholds of adjusted P-value (adj.P.val) < 0.05 and |log_2_ fold change| > 1.3 were applied. Visualization of the results was conducted through volcano plots and heatmaps generated via the ImageGP online platform (https://www.bic.ac.cn/ImageGP/).

#### 2.1.2 Functional enrichment analysis

The identified DEGs were subjected to Kyoto Encyclopedia of Genes and Genomes (KEGG) pathway enrichment and Gene Ontology (GO) biological process (BP) enrichment analyses using the MicroBioinformatics platform (https://www.bioinformatics.com.cn/), with statistical significance defined as P < 0.05.

#### 2.1.3 Weighted gene co-expression network analysis

The WGCNA algorithm was applied to construct a gene co-expression network. Initially, gene expression correlations were computed to generate a similarity matrix. Subsequently, hierarchical clustering combined with dynamic tree cutting algorithms was employed to delineate gene modules, with a minimum module size of 30 and sensitivity parameter set to 3. Evaluation of network topology across a range of soft-thresholding powers (β = 1 to 30) identified β = 10 as optimal for constructing a scale-free network (R² = 0.86); this value was selected as the lowest power at which the scale-free topology fit index exceeded 0.85, following the standard criterion recommended by the WGCNA package. Modules with similar expression profiles were subsequently merged using a height cutoff of 0.25 in the hierarchical clustering dendrogram. Correlations between module eigengenes and BPD status were calculated to determine modules significantly associated with the disease.

#### 2.1.4 Core gene identification and validation

Aging-related genes were sourced from the Aging Atlas database (https://ngdc.cncb.ac.cn/aging/index). Intersection analysis among DEGs, key module genes identified by WGCNA, and aging-related genes was performed using an online Venn diagram tool (https://bioinformatics.psb.ugent.be/webtools/Venn/) to pinpoint core genes. The diagnostic performance of these core genes was assessed by generating receiver operating characteristic (ROC) curves and calculating the area under the curve (AUC) values. Furthermore, gene set enrichment analysis (GSEA) based on KEGG pathways was conducted to elucidate the biological functions of the core genes.

#### 2.1.5 Protein interaction prediction and molecular docking

Protein-protein interactions between Noggin (NOG) and members of the bone morphogenetic protein (BMP) family were predicted using the STRING database (version 11.5, https://string-db.org/), with interaction confidence evaluated by combined scores. Three-dimensional structures of Noggin and BMP4 proteins were obtained from the AlphaFold protein structure database (https://alphafold.ebi.ac.uk/). Prior to docking, energy minimization of both protein structures was performed using the GROMACS force field to relieve steric clashes. Protein-protein rigid docking was performed via the ZDOCK online server (https://zdock.wenglab.org/), retaining the top ten docking conformations. Detailed interface analyses, including interface area, hydrogen bond count, salt bridge interactions, and binding free energy (ΔG) calculations, were conducted using the PDBePISA tool (https://www.ebi.ac.uk/msd-srv/prot_int/). The optimal docking conformation was selected based on the highest ZDOCK score combined with evaluation of interface quality, including the number of hydrogen bonds, salt bridges, and interface area, as assessed by PDBePISA. Visualization of the Noggin–BMP4 complex, key binding residues, and interaction patterns was accomplished using PyMOL software.

### 2.2 Cell-based experiments

#### 2.2.1 Cell culture and hyperoxia model development

Human pulmonary microvascular endothelial cells (HPMECs) were procured from Xiamen Yimo Biotechnology Co., Ltd. (Catalog No.: IM-H659). All experimental procedures were conducted using cells up to the 10th passage. The cells were allocated into two groups: normoxic (21% O_2_) and hyperoxic (85% O_2_). To establish a BPD cellular model, the hyperoxic group was continuously exposed to an atmosphere of 85% O_2_, 5% CO_2_, and 10% N_2_ within a dedicated incubator for 48 hours. Oxygen concentration was continuously monitored throughout the exposure period using a built-in oxygen sensor.

#### 2.2.2 Small interfering RNA transfection

siRNA transfection was performed in accordance with the manufacturer’s protocol for the Lipo8000™ transfection reagent. Four experimental groups were established: normoxia with negative control siRNA (siNC), normoxia with siRNA targeting Noggin (siNOG), hyperoxia with siNC, and hyperoxia with siNOG. The siRNA sequences were synthesized by Shanghai Sangon Biotech Co., Ltd. (**Supplementary Table 1**). Twenty-four hours following transfection, cells were subjected to a 48-hour treatment consistent with the experimental design.

#### 2.2.3 Assessment of cellular senescence

Cells were washed twice with phosphate-buffered saline (PBS), followed by fixation with 1 ml of fixation solution at room temperature for 15 minutes. After three additional PBS washes, 1 ml of staining working solution—prepared according to the kit instructions—was added, and cells were incubated overnight at 37 °C in a CO_2_-free incubator. Cellular senescence was subsequently observed and documented using an optical microscope. To quantify the extent of cellular senescence, the acquired images were analyzed using ImageJ software. For each group, three randomly selected independent fields (n = 3) were evaluated. The positive blue signals were isolated by applying a uniform threshold, and the proportion of the SA-β-gal positive staining area relative to the total field area (%Area) was calculated.

#### 2.2.4 Quantitative real-time polymerase chain reaction

Total RNA was extracted employing the TRIzol reagent method. RNA concentration and purity were evaluated spectrophotometrically, ensuring an A260/A280 ratio between 1.8 and 2.0. One microgram of RNA was reverse transcribed into complementary DNA (cDNA) using a reverse transcription kit. Quantitative real-time PCR was conducted utilizing the SYBR Green detection method in a 20 μl reaction volume. Relative gene expression levels were calculated using the 2^(-ΔΔCt) method, with β-actin serving as the internal reference gene. Primer sequences are provided (**Supplementary Tables 2**, **3**). Each sample was analyzed in triplicate technical replicates, and the entire experiment was independently repeated three times.

#### 2.2.5 Western blot analysis

Cells were lysed in RIPA buffer supplemented with protease inhibitors and homogenized thoroughly on ice for 30 minutes. The lysates were centrifuged at 12,000 rpm for 15 minutes at 4 °C, and the supernatants were collected. Protein concentrations were determined using the bicinchoninic acid (BCA) assay. Samples were mixed with 5× SDS loading buffer and denatured at 100 °C for 10 minutes. Thirty micrograms of protein per sample were separated by 10% SDS-PAGE and transferred onto polyvinylidene difluoride (PVDF) membranes pre-activated with methanol via wet transfer. Membranes were blocked with 5% nonfat dry milk at room temperature for one hour, followed by three washes with TBST. Primary antibodies were incubated overnight at 4 °C at the following dilutions: anti-Noggin (Abcam, ab16054, 1:1000), anti-BMP4 (Abcam, ab124715, 1:5000), anti-p53 (Proteintech, 10442-1-AP, 1:5000), anti-p21 (Proteintech, 10335-1-AP, 1:1000), and anti-β-tubulin (Absin, abs830032, 1:10000). After three additional TBST washes, membranes were incubated with HRP-conjugated goat anti-mouse IgG (Beyotime, A0216, 1:2000) or HRP-conjugated goat anti-rabbit IgG (H+L) (Beyotime, A0208, 1:2000) at room temperature for one hour, followed by three further washes. Protein bands were visualized using enhanced chemiluminescence (ECL) substrate and imaged with a chemiluminescence detection system. Band intensities were normalized to β-tubulin as the loading control. Densitometric analysis was performed using ImageJ software.

#### 2.2.6 Immunofluorescence staining

Cells were cultured to 60–80% confluence, washed three times with PBS, and fixed with 4% paraformaldehyde at room temperature for 15 minutes. Permeabilization was conducted using 0.3% Triton X-100 for 10 minutes at room temperature, followed by blocking with 5% goat serum for one hour. Primary antibodies against p53 and p21 (both at 1:200 dilution) were applied and incubated overnight at 4 °C. After three PBS washes, cells were incubated with fluorescently labeled secondary antibodies for one hour at room temperature in the dark. Following three additional washes, nuclei were counterstained with DAPI for five minutes. Samples were mounted with anti-fade mounting medium and imaged using a laser scanning confocal microscope. Fluorescence intensity quantification was performed using ImageJ software.

#### 2.2.7 Enzyme-linked immunosorbent assay

Cell culture supernatants were collected and centrifuged at 3,000 rpm for 10 minutes at 4 °C to remove cellular debris. Concentrations of Noggin and BMP4 proteins were measured using commercially available ELISA kits (Noggin: MLBIO, Cat. No. ml6091H-2; BMP4: MLBIO, Cat. No. ml106427) according to the manufacturer’s protocols. Samples were diluted as recommended in the kit instructions prior to measurement. Each sample was assayed in triplicate. Absorbance was read at 450 nm, and protein concentrations were calculated based on the standard curve.

### 2.3 Animal experiments

#### 2.3.1 Experimental animals

Specific pathogen-free (SPF) grade Sprague-Dawley rats were obtained from the Experimental Animal Center of Hubei University of Medicine. Neonatal pups born within a 12-hour interval were used to minimize developmental variability. Animals were housed under standard laboratory conditions (temperature 20–22 °C, humidity 55–65%, 12-hour light/dark cycle) with ad libitum access to food and water.

#### 2.3.2 Establishment of BPD animal model

Newborn pups, within 24 hours of birth, were randomly allocated to either the control or model groups. The control group was housed in normoxic conditions (21% oxygen), whereas the model group was continuously exposed to a hyperoxic environment with 85% oxygen concentration. Oxygen levels were precisely regulated at 85 ± 1% using an oxygen generator with real-time monitoring. Carbon dioxide concentration was maintained below 0.5% through regular replacement of sodium lime. To mitigate oxygen toxicity, cages were opened for 30 minutes every 24 hours, during which nursing dams were rotated between groups. Pups were euthanized at postnatal days 1, 7, 14, and 21 for tissue collection.

#### 2.3.3 Specimen collection and processing

Pups were anesthetized via inhalation of isoflurane. Following disinfection with 75% ethanol, the thoracic cavity was surgically opened. Lungs were perfused through the right ventricle with ice-cold phosphate-buffered saline (PBS; 5–20 ml) until the lung tissue appeared blanched. The main bronchi were dissected and ligated above the tracheal bifurcation, and both lungs were excised intact. The left lung was fixed in 4% paraformaldehyde for 24 hours for subsequent hematoxylin and eosin (HE) staining. The right lung was sectioned, rapidly frozen in liquid nitrogen, and stored at –80 °C for molecular biological analyses.

#### 2.3.4 Histological examination

Fixed lung tissues were routinely processed, paraffin-embedded, and cut into 5-μm sections. Following deparaffinization and rehydration, sections were stained with hematoxylin and eosin (H&E) and examined under an optical microscope. For morphometric quantification of alveolar simplification, the mean linear intercept (MLI) was determined using ImageJ software. A cross-grid was superimposed on randomly selected, non-overlapping fields (excluding large airways and blood vessels), and MLI was calculated by dividing the total grid line length by the number of intersections with alveolar septa.

#### 2.3.5 RNA extraction from tissue

Lung tissues were pulverized under liquid nitrogen. Total RNA was extracted using the TRIzol reagent according to the manufacturer’s protocol. Subsequent quantitative real-time PCR (qRT-PCR) procedures were conducted following the same protocol as described for the cell-based experiments.

### 2.4 Statistical analysis

Data were analyzed using GraphPad Prism version 10. Prior to statistical testing, normality of data distribution was assessed using the Shapiro–Wilk test, and variance homogeneity was evaluated using Levene’s test before applying ANOVA. Quantitative results are presented as mean ± standard deviation (mean ± SD). Comparisons between two groups were performed using independent samples t-tests. For comparisons involving multiple groups, one-way analysis of variance (ANOVA) followed by Tukey’s *post hoc* test was employed. A p-value less than 0.05 was considered indicative of statistical significance. All cell-based experiments were independently replicated three times, with three technical replicates per experiment.

## 3 Results

### 3.1 Differential gene expression analysis reveals immune-related pathway enrichment in BPD

**Figure 1:**
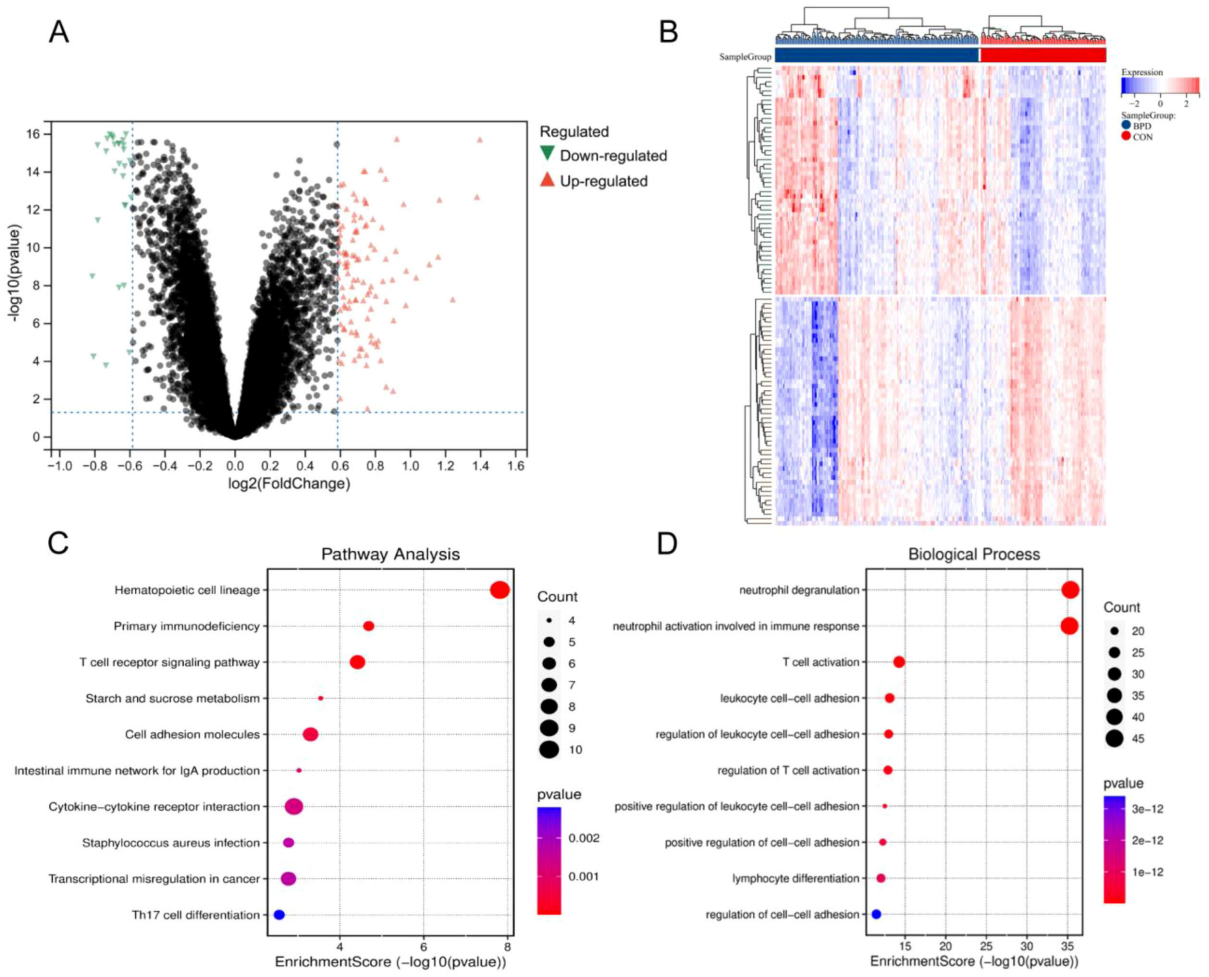
Differential gene expression analysis reveals immune dysregulation molecular signature in BPD. **(A)** Volcano plot displaying differentially expressed genes (DEGs) between BPD patients and healthy controls from GSE32472 dataset. Red dots represent upregulated genes (n=114), blue dots represent downregulated genes (n=51), and gray dots represent non-significant genes. Selection criteria: adj.P.val < 0.05 and |log2FC| > 1.3. **(B)** Heatmap showing hierarchical clustering of DEGs expression patterns. **(C)** KEGG pathway enrichment analysis of DEGs. **(D)** Gene Ontology biological process (GO-BP) enrichment analysis of DEGs. Top enriched terms are shown. Statistical significance was determined by adjusted P value < 0.05.

To systematically elucidate the molecular pathological mechanisms of BPD, we obtained the GSE32472 dataset from the GEO database for in-depth bioinformatics analysis. This dataset contains peripheral blood transcriptome expression profiles from children with BPD and healthy controls. Differential expression analysis was performed using the limma package in R, with adjusted p-value (adj.P.val) < 0.05 and |log2 fold change (FC)| > 1.3 as the screening criteria. A total of 165 differentially expressed genes (DEGs) were identified, including 114 upregulated and 51 downregulated genes. The volcano plot clearly displayed the distribution pattern of DEGs, with significantly upregulated and downregulated genes located on opposite sides of the plot, demonstrating distinct expression differences (**Figure 1A**). Hierarchical clustering heatmap further revealed significant differentiation in DEG expression patterns between the two groups, with visually distinct clustering patterns between the BPD and control groups as assessed from the heatmap, suggesting that these DEGs may effectively distinguish disease status (**Figure 1B**).

To further interpret the biological significance of these DEGs, we conducted systematic functional enrichment analyses. KEGG pathway enrichment analysis showed that DEGs were significantly enriched in multiple immune response-related signaling pathways, mainly including hematopoietic cell lineage, primary immunodeficiency, and T cell receptor signaling pathways (**Figure 1C**). GO biological process (BP) enrichment analysis further confirmed that these DEGs are primarily involved in key biological processes of innate and adaptive immune responses, such as neutrophil degranulation, neutrophil activation involved in immune response, and T cell activation (**Figure 1D**). These enrichment results suggest that immune-related pathways may contribute to the pathogenesis of BPD.

### 3.2 WGCNA and aging database integration identify Noggin as a hub gene linking BPD and senescence

**Figure 2:**
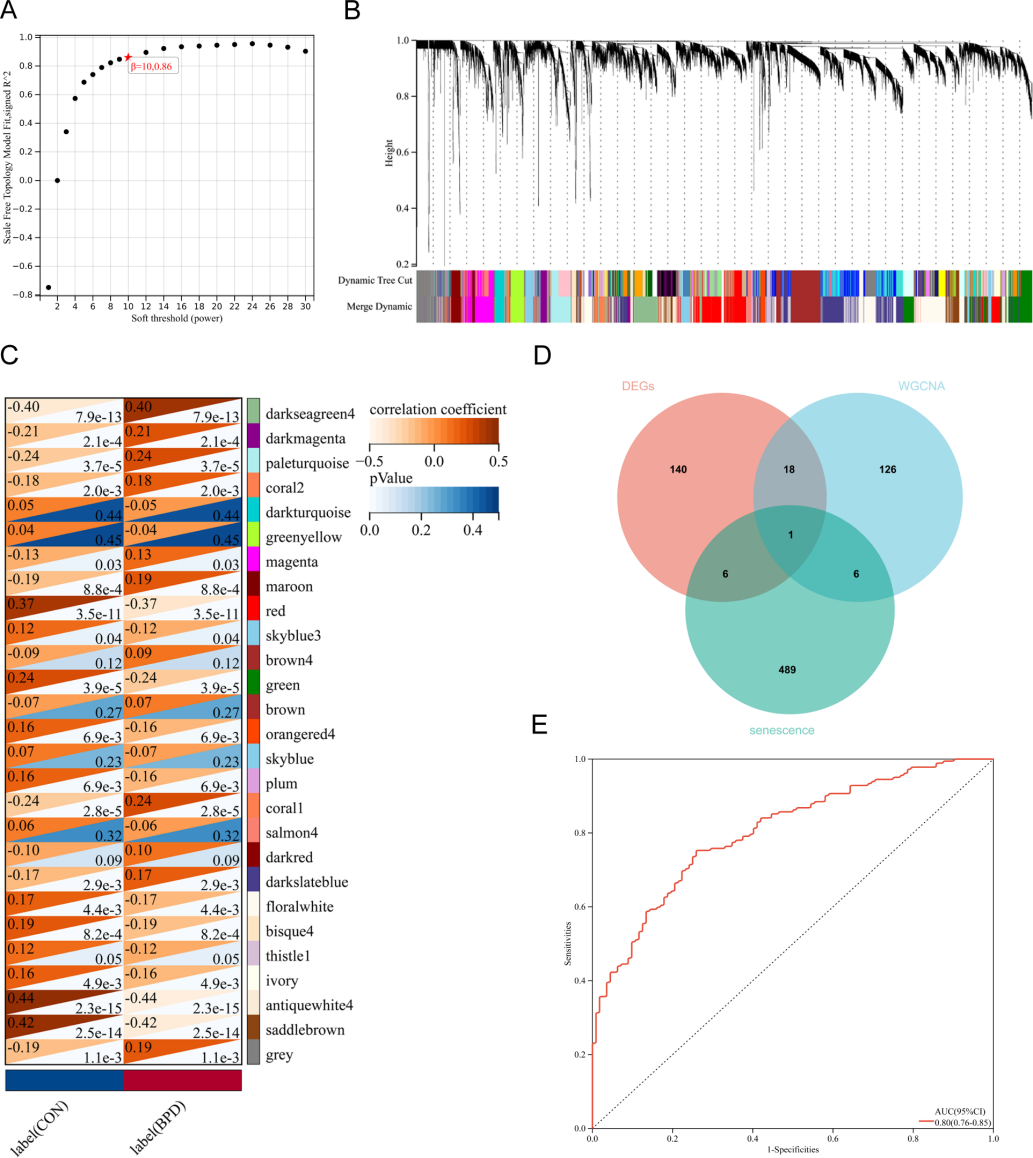
WGCNA network analysis and multi-dataset integration identify Noggin as the key hub gene. **(A)** Scale-free topology fitting index (R²) for soft-thresholding powers. β=10 was selected (R²=0.86). **(B)** Dendrogram showing gene clustering and 27 color-coded modules. **(C)** Module-trait correlation heatmap. Antiquewhite4 module showed strongest correlation with BPD. **(D)** Venn diagram of DEGs, antiquewhite4 module genes, and aging-related genes. Noggin was the only overlapping gene. **(E)** ROC curve of NOG expression. AUC = 0.80 (95% CI: 0.76-0.85).

To identify core gene modules closely associated with BPD pathogenesis from a systems biology perspective, we performed WGCNA on the GSE32472 dataset. By systematically evaluating the topological characteristics of the network under different soft-thresholding powers (β values from 1 to 30), we ultimately selected β = 10 as the optimal parameter to construct a scale-free co-expression network. At this threshold, the scale-free topology fit index R² reached 0.86, meeting the criteria for a scale-free network (R² > 0.85), while maintaining a moderate average connectivity (**Figure 2A**). Based on gene expression similarity measures and the topological overlap matrix (TOM), hierarchical clustering and dynamic tree cutting algorithms divided all genes into 27 relatively independent co-expression modules, each labeled with a specific color (**Figure 2B**). Module-phenotype correlation analysis showed that the darkseagreen4 module had the strongest positive correlation with BPD disease status, indicating that genes within this module may play a core regulatory role in BPD pathogenesis (**Figure 2C**).

To further pinpoint key targets associated with both BPD and cellular senescence, we employed a multi-dataset cross-validation strategy. We intersected the 165 DEGs from differential expression analysis, key module genes identified by WGCNA, and senescence marker genes collected from the Aging Atlas database using a Venn diagram. The results showed that only the NOG gene formed a unique intersection among the three datasets, indicating that NOG is a core candidate gene that simultaneously meets the criteria of differential expression in BPD, membership in disease-related modules, and involvement in senescence regulation (**Figure 2D**). To evaluate the clinical diagnostic value of NOG as a potential biomarker for BPD, we extracted its expression values from all samples in the GSE32472 dataset and plotted the receiver operating characteristic (ROC) curve. The analysis showed that the area under the curve (AUC) for NOG was 0.80 (95% CI: 0.76–0.85), indicating that its expression level has good diagnostic ability to distinguish children with BPD from healthy controls (**Figure 2E**). To further explore the biological functions and signaling pathways potentially involving NOG, we conducted single-gene gene set enrichment analysis (GSEA). GSEA-KEGG pathway analysis revealed that samples with high NOG expression were significantly enriched in pathways such as selenocompound metabolism, cysteine and methionine metabolism, intestinal immune network for IgA production, cell adhesion molecules, ribosome, and circadian rhythm (**Supplementary Figure S1**). These findings suggest that NOG may participate in the complex pathophysiological processes of BPD through multidimensional regulatory mechanisms.

### 3.3 Hyperoxia-induced senescence phenotypes in HPMECs

**Figure 3:**
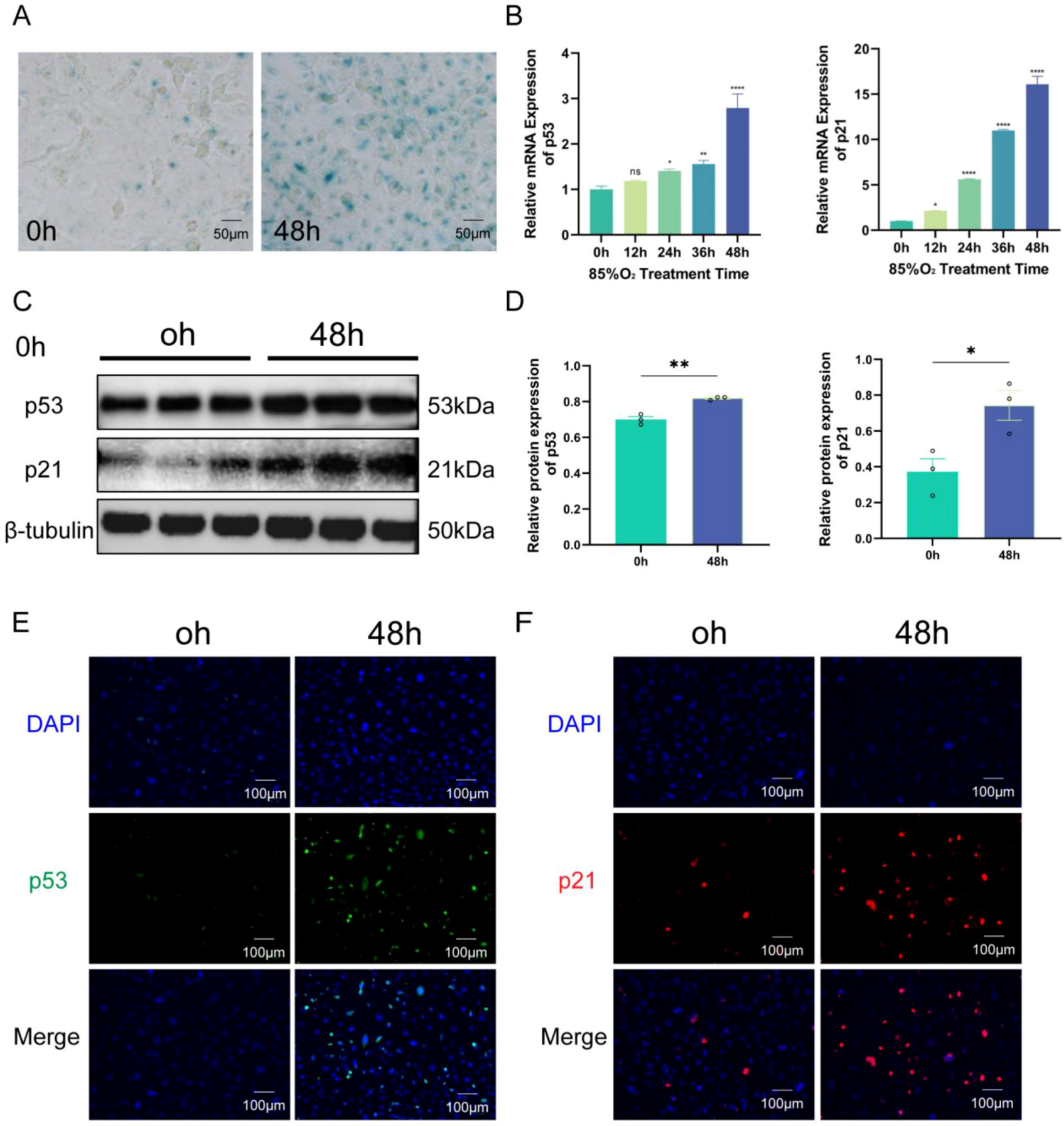
Hyperoxia induces senescence phenotype in HPMECs. **(A)** Phase-contrast and SA-β-gal staining of HPMECs exposed to normoxia (21% O_2_, 0h) or hyperoxia (85% O_2_, 48h) for 48h. Blue indicates senescent cells. Scale bar=50 μm. **(B)** qRT-PCR of p53 and p21 mRNA at 0, 12, 24, 36, and 48h hyperoxia. Data: mean ± SD (n=3). ns means not significant; *P < 0.05, **P < 0.01, ****P < 0.0001 vs. 0h. **(C, D)** Western blot and quantification of p53 and p21 proteins after 48h treatment. β-tubulin as loading control. *P< 0.05, **P<0.01 vs. normoxia. **(E, F)** Immunofluorescence and quantification of p53 (green) and p21 (red). DAPI (blue) for nuclei. Scale bar=100 μm. Data: mean ± SD (n=3).

To investigate the relationship between bronchopulmonary dysplasia (BPD) and cellular senescence, we developed an *in vitro* model of hyperoxia-induced injury using human pulmonary microvascular endothelial cells (HPMECs). Cells were subjected to 85% oxygen for 48 hours, while normoxic conditions (21% oxygen) served as controls. Morphological assessment via optical microscopy revealed that hyperoxia-exposed HPMECs exhibited hallmark features of senescence, including markedly increased cell size with a flattened and spread morphology, enlarged nuclei displaying nuclear membrane invaginations, and enlarged nucleoli (**Figure 3A**). To further substantiate the induction of senescence, senescence-associated β-galactosidase (SA-β-gal) staining, a widely accepted marker for senescent cells, was performed. Hyperoxia-treated cells demonstrated extensive cytoplasmic blue staining indicative of SA-β-gal activity. Quantitative analysis revealed that the proportion of the SA-β-gal positive staining area was significantly increased in the hyperoxia-exposed group (21.67%) compared to the normoxic controls (0.31%) (p < 0.0001), which showed minimal staining (**Figure 3A**; **Supplementary Figure S2**). At the molecular level, we assessed the expression of canonical senescence markers p53 and p21. Quantitative real-time PCR (qRT-PCR) analysis revealed a time-dependent upregulation of p53 and p21 mRNA expression following hyperoxia exposure, with maximal expression observed at 48 hours (**Figure 3B**). Consistently, Western blot analysis confirmed significant increases in p53 and p21 protein levels after 48 hours of hyperoxia relative to normoxic controls (**Figures 3C, D**). Immunofluorescence staining further demonstrated enhanced nuclear localization and expression intensity of p53 (green) and p21 (red) in hyperoxia-treated cells, with nuclei counterstained by DAPI (**Figures 3E, F**). Collectively, these multi-faceted experimental findings—including morphological changes, SA-β-gal staining, and gene and protein expression analyses—validate the establishment of a robust hyperoxia-induced senescence model in HPMECs. This model effectively recapitulates endothelial cell aging processes relevant to BPD pathogenesis and highlights the prominent activation of the classical p53/p21 senescence signaling pathway during hyperoxia-induced cellular aging.

### 3.4 Noggin directly antagonizes BMP4 and hyperoxia disrupts their expression balance

**Figure 4:**
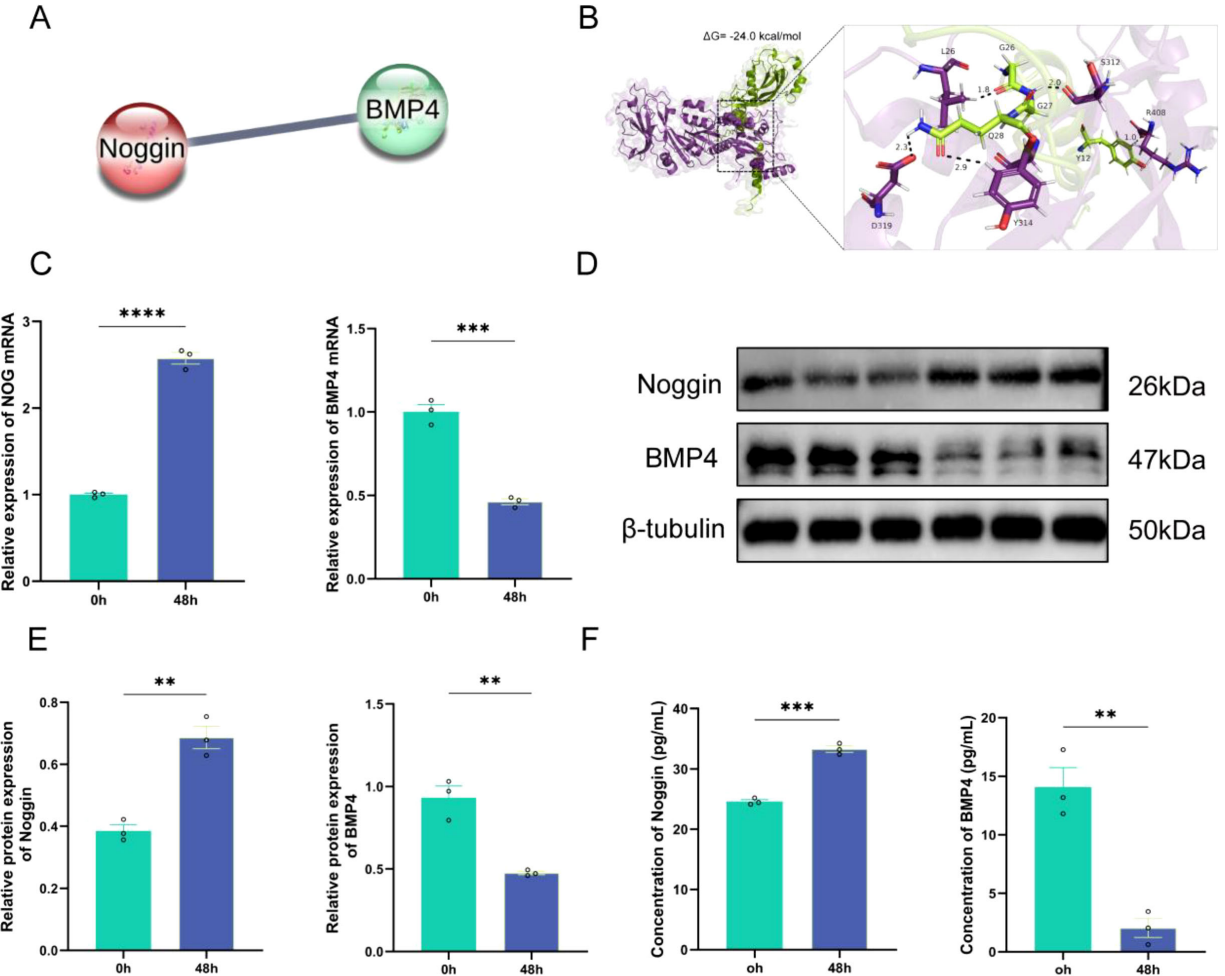
Noggin directly interacts with BMP4 and shows expression imbalance under hyperoxia. **(A)** STRING database prediction of Noggin-BMP4 protein interactions. **(B)** Molecular docking of Noggin-BMP4 (ΔG= -24.0 kcal/mol). **(C)** qRT-PCR of NOG and BMP4 mRNA after 48h normoxia/hyperoxia. **(D, E)** Western blot of Noggin and BMP4 proteins. β-tubulin as loading control. **(F)** ELISA of Noggin and BMP4 in culture supernatants. Data: mean ± SD (n=3). **P < 0.01, ***P < 0.001, ****P < 0.0001 vs. normoxia.

Following the identification of NOG as a key gene through prior screening, we further investigated its potential mechanistic roles. The Noggin protein, encoded by NOG, functions as a critical antagonist within the bone morphogenetic protein (BMP) signaling pathway by binding BMP ligands and thereby inhibiting their interaction with BMP receptors. To systematically assess the interaction between Noggin and members of the BMP family, we initially conducted predictive analyses utilizing the STRING database. The results suggested a direct protein-protein interaction between Noggin and BMP4, characterized by a high interaction score, indicating a likely close biological relationship between these proteins (**Figure 4A**). To explore the molecular basis of this interaction, molecular docking simulations were performed to model the binding conformation of Noggin and BMP4. The docking simulation suggested a stable binding interaction between Noggin and BMP4, with the resulting complex displaying a calculated binding free energy (ΔG) of -24.0 kcal/mol. This low energy value indicates a strong predicted binding affinity between the two proteins (**Figure 4B**).

Guided by these predictive insights, we subsequently examined the expression dynamics of Noggin and BMP4 in hyperoxia-induced HPMECs. Quantitative real-time PCR (qRT-PCR) analysis indicated that after 48 hours of hyperoxia exposure, NOG mRNA expression was significantly upregulated by approximately 2.5-fold (P < 0.001) relative to normoxic controls, whereas BMP4 mRNA expression was markedly downregulated to approximately 50% of control levels (P < 0.001), demonstrating inverse expression patterns (**Figure 4C**). Western blot analyses corroborated these findings at the protein level, revealing an approximate twofold increase in Noggin protein expression and a concomitant reduction of BMP4 protein to about 50% of control levels in hyperoxia-treated cells (**Figure 4D**).Given that both Noggin and BMP4 are secreted proteins, alterations in their extracellular concentrations may more directly reflect their functional activity. Therefore, enzyme-linked immunosorbent assays (ELISA) were employed to quantify Noggin and BMP4 protein levels in cell culture supernatants. Under normoxic conditions, Noggin concentration averaged approximately 24 pg/mL, which significantly increased to approximately 33 pg/mL following 48 hours of hyperoxia exposure, representing a 1.4-fold elevation (P < 0.001). Conversely, BMP4 concentration decreased from approximately 14 pg/mL under normoxia to about 2 pg/mL post-hyperoxia, corresponding to a sevenfold reduction (P < 0.01). These extracellular protein concentration changes were consistent with the intracellular mRNA and protein expression trends (**Figure 4E**). Collectively, these findings confirm a direct molecular interaction between Noggin and BMP4. Moreover, in the hyperoxia-induced bronchopulmonary dysplasia (BPD) cell model, the Noggin-BMP4 signaling axis exhibits significant dysregulation, suggesting that upregulation of Noggin may contribute to the modulation of cellular senescence through antagonism of BMP4 signaling.

### 3.5 Noggin silencing reverses hyperoxia-induced senescence by restoring BMP4 signaling

**Figure 5:**
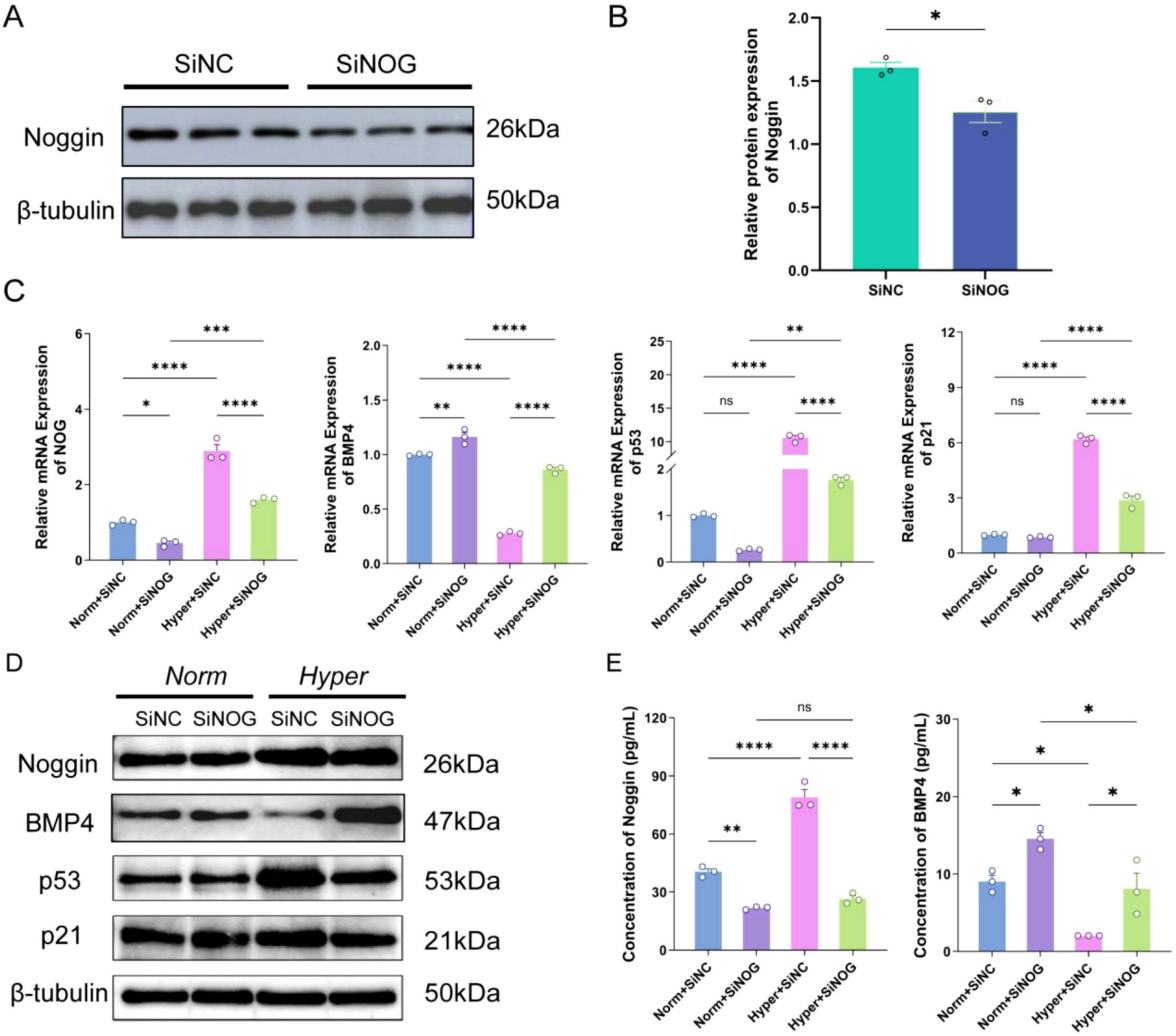
NOG silencing alleviates hyperoxia-induced senescence via BMP4 activation. **(A, B)** Western blot confirming Noggin knockdown efficiency at 24h post-transfection. siNC as control. **(C)** qRT-PCR of NOG, BMP4, p53, and p21 in four groups: Norm+siNC, Norm+siNOG, Hyper+siNC, Hyper+siNOG (48h treatment). **(D)** Western blot of Noggin, BMP4, p53, and p21. β-tubulin as loading control. **(E)** ELISA of Noggin and BMP4 in supernatants. Data: mean ± SD (n=3). ns means not significant; *P < 0.05, **P < 0.01, ***P < 0.001, ****P < 0.0001 vs. respective controls.

To elucidate the functional role of Noggin in hyperoxia-induced cellular senescence, we employed small interfering RNA (siRNA) technology to specifically suppress Noggin expression in HPMECs. Initially, the efficiency of siRNA-mediated knockdown was validated. Analysis demonstrated a significant reduction in NOG mRNA levels following transfection with the specific siRNA sequence SiNOG3 compared to the negative control (SiNC), achieving an interference efficiency exceeding 70%, thereby satisfying the criteria for subsequent functional assays (**Supplementary Figure S3**). Furthermore, Western blot analysis demonstrated a significant reduction in Noggin protein levels 24 hours post-transfection with this specific siRNA, thereby satisfying the criteria for subsequent functional assays (**Figures 5A, B**).

Following successful Noggin silencing, four experimental groups were established: normoxia control (Norm+siNC), normoxia with Noggin knockdown (Norm+siNOG), hyperoxia model (Hyper+siNC), and hyperoxia combined with Noggin silencing (Hyper+siNOG), each subjected to 48 hours of treatment. Quantitative real-time PCR (qRT-PCR) analysis confirmed the mRNA expression profiles of NOG and BMP4 across these groups. Results indicated that siNOG treatment significantly decreased NOG mRNA levels under both normoxic and hyperoxic conditions relative to controls, confirming effective gene silencing (**Figure 5C**). Importantly, BMP4 mRNA expression was markedly upregulated following Noggin knockdown in both oxygen conditions. Given that Noggin primarily functions as an extracellular antagonist that binds BMP ligands, this observed increase in BMP4 transcription likely represents an indirect regulatory feedback mechanism rather than direct transcriptional repression by Noggin. Assessment of senescence-associated markers p53 and p21 revealed that hyperoxia alone induced a substantial increase in p53 mRNA (~10-fold) and p21 mRNA (~6-fold) compared to normoxic controls (P < 0.001). Notably, co-treatment with siNOG under hyperoxic conditions significantly attenuated these elevations, reducing p53 and p21 mRNA levels by approximately 5-fold and 2-fold, respectively, relative to hyperoxia alone (P < 0.001). Although these levels remained elevated compared to normoxia controls, the reductions indicate a pronounced mitigation of senescence. Conversely, Noggin silencing under normoxia did not significantly alter baseline p53 and p21 expression, implying that Noggin’s modulatory effects on senescence are contingent upon hyperoxic stress. Western blot analyses corroborated these transcriptional findings at the protein level (**Figure 5D**). Noggin protein expression was significantly diminished following siNOG treatment, concomitant with an upregulation of BMP4 protein. In the hyperoxia plus siNOG group, BMP4 protein levels were restored to near normoxic control values. Furthermore, the hyperoxia-induced increases in p53 and p21 protein expression were substantially reduced by Noggin silencing, consistent with mRNA data. Enzyme-linked immunosorbent assay (ELISA) quantification of culture supernatants further substantiated these observations: siNOG treatment decreased Noggin protein concentration by approximately 50%, while BMP4 protein concentrations increased by approximately 1.6-fold and 4-fold under normoxic and hyperoxic conditions, respectively. These results confirm that Noggin silencing effectively reinstates BMP4 secretion suppressed by hyperoxia (**Figure 5E**).

### 3.6 Hyperoxia-induced BPD model recapitulates alveolar simplification and Noggin-BMP4 dysregulation

**Figure 6:**
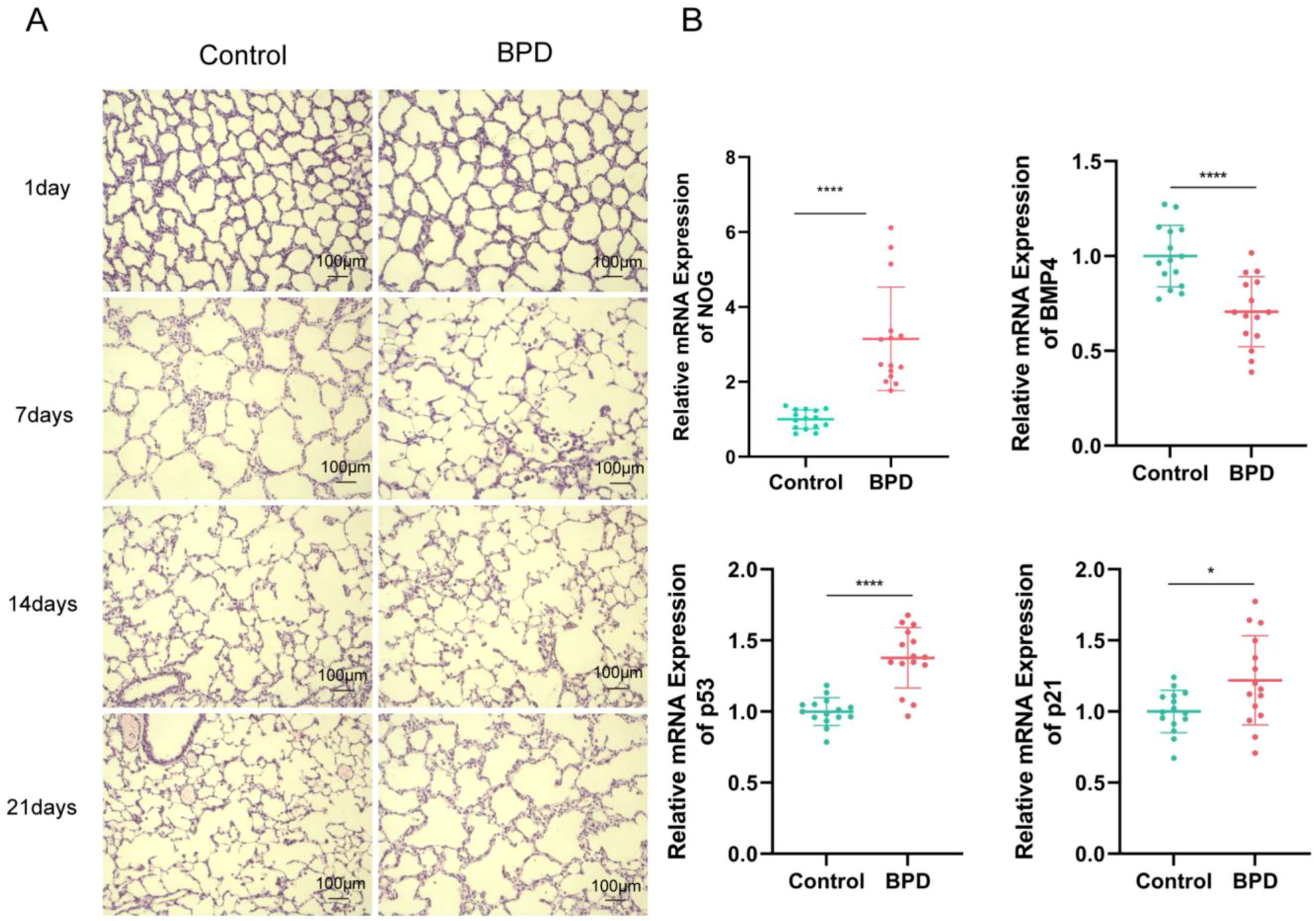
NOG-BMP4-p53/p21 axis imbalance in neonatal rat BPD model. **(A)** H&E staining of lung tissues at postnatal days 1, 7, 14, and 21. Control: normoxia (21% O_2_); Model: hyperoxia (85% O_2_). Scale bar=100 μm (n=5 per group). **(B)** qRT-PCR of NOG, BMP4, p53, and p21 at day 21. Data: mean ± SD (n=15 per group). *P<0.05, ****P<0.0001 vs. Control.

To elucidate the involvement of Noggin in the pathogenesis of BPD at the organismal level, a neonatal rat model subjected to hyperoxic exposure was established to simulate BPD. Lung tissue specimens were harvested at postnatal days 1, 7, 14, and 21 for comprehensive histopathological and molecular analyses. HE staining revealed the characteristic pathological progression associated with BPD. Lung tissues from control animals maintained normal alveolar architecture throughout all examined time points, characterized by uniform alveolar dimensions, thin alveolar septa, and well-defined cristae-like structures. In contrast, the hyperoxia-exposed group exhibited early pathological changes, including thickening of the alveolar septa beginning at day 7. Prolonged hyperoxic exposure led to a significant increase in alveolar volume by day 14, accompanied by irregular alveolar morphology. By day 21, pathological deterioration was evident, with adjacent alveoli merging into bullous formations, a pronounced decrease in alveolar number, progressive reduction or complete loss of the previously abundant cristae, and further septal thickening coupled with inflammatory cell infiltration. To quantitatively confirm these histological observations at the critical study endpoint, morphometric analysis was performed by measuring the mean linear intercept (MLI) of lung tissues at postnatal day 21. Consistent with the qualitative findings of alveolar enlargement, the MLI was significantly increased in the hyperoxia-exposed group compared to the normoxia control group (41.46 vs. 30.76 μm, P < 0.0001; **Supplementary Figure S4**). These histopathological features closely recapitulate the alveolar simplification observed in lung tissues from patients clinically diagnosed with BPD (**Figure 6A**).

To further investigate the role of the Noggin-BMP4 signaling axis and cellular senescence in BPD pathogenesis, lung tissues collected at day 21 were subjected to qRT-PCR analysis to assess the expression levels of key genes. Compared to controls, the hyperoxia-exposed group demonstrated a significant upregulation of NOG mRNA expression, approximately threefold higher (P < 0.001). Conversely, BMP4 mRNA levels were markedly decreased to about 30% of control values (P < 0.001). Concurrently, mRNA expression of senescence-associated markers p53 and p21 were elevated by approximately 1.4-fold (P < 0.001) and 1.2-fold (P < 0.05), respectively (**Figure 6B**). These *in vivo* results are consistent with the molecular expression patterns previously observed in the hyperoxia-induced BPD cellular model, thereby reinforcing the proposed mechanistic involvement of the Noggin-BMP4-p53/p21 signaling axis in BPD pathogenesis.

## 4 Discussion

This study suggests that Noggin may function as an important upstream regulator promoting senescence in pulmonary microvascular endothelial cells under hyperoxic conditions and supports the involvement of the dysregulated Noggin–BMP4–p53/p21 signaling axis in the pathogenesis of BPD. Utilizing an integrated approach comprising bioinformatics analyses, *in vivo* animal models, and *in vitro* functional assays with HPMECs, we systematically show that hyperoxia exposure is associated with sustained overexpression of Noggin. This overexpression antagonizes BMP4 signaling, thereby activating p53/p21-mediated cellular senescence. This mechanism not only compromises pulmonary vascular development but may also disrupt essential angiocrine signaling, ultimately leading to arrested alveolar development. These findings address a critical gap in understanding the molecular interplay between oxidative stress, endothelial injury, and alveolar simplification, offering a novel perspective for investigating BPD pathogenesis and developing targeted therapeutic strategies.

At the molecular level, this study further explores the role of the Noggin-BMP4 signaling axis in hyperoxia-induced cellular senescence. Structural biology evidence indicates that Noggin forms a high-affinity complex with BMP4 via its cystatin domain. Molecular docking analysis suggested a potentially stable binding interaction between Noggin and BMP4. This binding affinity suggests that, under hyperoxic pathological conditions, elevated Noggin levels can effectively inhibit BMP4 binding to its type II receptor, thereby suppressing downstream Smad1/5/8 phosphorylation and nuclear translocation (Phan-Everson et al., 2021). Under physiological conditions, BMP signaling is essential for maintaining pulmonary cellular homeostasis: the BMP4-Smad1/5/8 pathway regulates the balance between proliferation and differentiation in alveolar epithelial cells (Shen et al., 2020; Zheng et al., 2024), while ligands such as BMP9 and BMP10 play pivotal roles in vascular endothelial development (Chen et al., 2013). Our findings suggest that sustained Noggin overexpression under hyperoxia is associated with prolonged inhibition of BMP4 signaling in endothelial cells, which may impair cellular proliferation and differentiation and activate p53/p21-mediated cell cycle arrest. This endothelial senescence functions as a protective mechanism against oxidative stress-induced vascular damage but concurrently compromises vascular regenerative capacity (Han and Kim, 2023).

From a systems biology perspective, the Noggin-BMP4 axis constitutes a complex regulatory signaling network intertwined with oxidative stress, endoplasmic reticulum (ER) stress, and inflammatory responses. This study provides a potential mechanistic explanation for the initially observed phenomenon of immune-related pathway enrichment identified via bioinformatics analyses. During the screening process, differentially expressed genes were notably enriched in immune-related pathways, including neutrophil degranulation and T cell activation. This finding closely corresponds with the biological characteristics of endothelial cell senescence. Senescent endothelial cells acquire a senescence-associated secretory phenotype (SASP), characterized by the release of pro-inflammatory mediators that sustain local inflammation and may contribute to the enrichment of immune-related pathways observed in our bioinformatics analysis (Sabbatinelli et al., 2019; Bloom et al., 2023). Our study shows that hyperoxia exposure significantly elevates NOG expression while concurrently suppressing BMP4 levels, a pattern consistently observed in both *in vivo* and *in vitro* models. Regarding the upstream regulatory mechanism of Noggin upregulation, it is established that reactive oxygen species (ROS) generated by hyperoxia can modulate target gene expression through activation of redox-sensitive transcription factors such as nuclear factor kappa B (NF-κB) and activator protein 1 (AP-1) (Morgan and Liu, 2011; Pepperl et al., 2001). We hypothesize that a similar mechanism may underlie NOG transcriptional activation. However, this proposition requires validation through promoter analyses and chromatin immunoprecipitation assays. At the downstream effector level, oxidative stress activates myeloperoxidase (MPO) via ER stress, establishing a positive feedback loop encompassing ROS, ER stress, and cellular senescence (Faraonio, 2022). Jing et al. demonstrated that treatment with tauroursodeoxycholic acid (TUDCA) or the MPO inhibitor KYC effectively mitigates cellular senescence in a BPD model (Jing et al., 2024). The findings suggest that the Noggin–BMP4 axis may represent an important regulatory link between oxidative stress and endothelial senescence.

Recent studies have underscored the centrality of cellular senescence in BPD pathogenesis, yet the upstream mechanisms initiating this process remain inadequately understood. Our findings substantially complement existing literature. For instance, Jing et al. employed single-cell RNA sequencing to analyze a hyperoxia-induced BPD rat model, confirming that multiple cell types, including AT2 cells, exhibit pronounced senescent phenotypes marked by elevated expression of senescence markers p16, p53, and Foxo4, with senescence induced by DNA damage rather than telomere attrition (Jing et al., 2024). Yao et al. utilized p21-CreER lineage tracing mice to demonstrate that neonatal hyperoxia-induced senescence manifests distinct temporal and cell-type specificity (Yao et al., 2023). Behnke et al. focused on mesenchymal stem cells derived from preterm infant lungs, revealing that mechanical stretch combined with hyperoxia induces p21-mediated senescence (Behnke et al., 2024). While these investigations primarily addressed senescence phenotypes and downstream consequences, our study uniquely traces upstream regulatory events, identifying Noggin as a critical factor initiating the senescence cascade.

Regarding BMP signaling regulation, our research delineates a fundamental distinction between physiological repair and pathological injury. Previous studies have demonstrated that, during normal lung repair, BMP signaling undergoes transient and dynamic modulation—briefly suppressed to permit progenitor cell proliferation before being restored to drive differentiation (Shen et al., 2020). In contrast, our findings indicate that hyperoxia exposure induces sustained high expression of Noggin, resulting in an aberrant “locked state” that causes prolonged suppression of BMP4 signaling. This persistent inhibition prevents endothelial cells from completing the normal proliferation-differentiation cycle, thereby inducing senescence. Consequently, the aberrant temporal regulation of this signaling pathway—shifting from “transient fluctuation” to “persistent inhibition”—constitutes a critical determinant distinguishing regenerative repair from fibrotic senescence.

Based on these observations, we propose that endothelial senescence in BPD arises not solely as a consequence of oxidative damage but from a “temporal dysregulation” of repair signaling. As discussed above, whereas physiological repair relies on transient and reversible BMP signal modulation, persistent hyperoxia disrupts this equilibrium by upregulating Noggin, leading to sustained BMP signaling suppression, which arrests endothelial cells at cell cycle checkpoints and ultimately activates p53/p21-mediated senescence pathways. This mechanism highlights that the “duration” rather than the “magnitude” of signaling perturbation determines irreversible damage, thereby explaining why brief exposure to high-concentration oxygen is relatively safe clinically, whereas prolonged moderate exposure predisposes to BPD. This framework integrates oxidative stress, signaling imbalance, inflammatory cascades, and vascular-alveolar crosstalk into a comprehensive pathway, suggesting a key transition from acute injury to chronic developmental disorder. Notably, such temporal dysregulation of BMP signaling may represent a common pathological feature across diverse tissue regeneration disorders. For example, Guan et al. demonstrated that downregulation of BMP4 signaling in idiopathic pulmonary fibrosis induces fibroblast senescence and mitochondrial autophagy defects, whereas enhancement of BMP4 signaling reverses the fibrotic phenotype (Guan et al., 2022). This suggests that targeting the Noggin-BMP4 axis may hold therapeutic potential for multiple chronic pulmonary diseases.

This study bears significant clinical translational implications. First, as a secreted protein, Noggin may represent a potential candidate biomarker, although validation in clinical cohorts is required. Our ELISA confirmed that Noggin protein concentrations in extracellular fluids markedly increase under hyperoxic conditions, consistent with intracellular expression patterns. This finding implies that quantification of Noggin levels in serum or airway lavage fluid from preterm infants could complement existing biomarkers (e.g., 8-hydroxy-2’-deoxyguanosine [8-OHdG], N-terminal pro-B-type natriuretic peptide [NT-proBNP]) (Iliodromiti et al., 2020; Cui and Fu, 2022), facilitating the development of multidimensional early risk prediction models. Second, the Noggin-BMP4 axis constitutes a promising therapeutic target. We showed that siRNA-mediated NOG silencing attenuates hyperoxia-induced senescent phenotypes, underscoring the therapeutic value of modulating this pathway. Nevertheless, given Noggin’s critical role in early lung branching morphogenesis and the dose sensitivity of BMP signaling (Wei et al., 2022; Li et al., 2023), future therapeutic strategies should prioritize lung vascular-specific targeted delivery or the development of small-molecule inhibitors that selectively disrupt Noggin-BMP4 interactions. Additionally, downstream senolytic approaches targeting Noggin-induced senescent cells, such as combined dasatinib and quercetin treatment (Fan et al., 2022; Nambiar et al., 2023), may enhance the vascular microenvironment by eliminating senescent endothelial cells, thus promoting secondary alveolar development.

Several limitations warrant consideration. First, although siRNA-mediated NOG silencing ameliorates the senescent phenotype, reciprocal experiments involving Noggin overexpression and exogenous BMP4 supplementation were not conducted, and the BMP4 dose-response relationship remains uncharacterized. These gaps limit comprehensive elucidation of the Noggin-BMP4 axis regulatory mechanisms. Second, while hyperoxia exposure alone constitutes a classical BPD paradigm, human BPD typically involves multifactorial insults, including infection and malnutrition. This study lacks direct evidence derived from human clinical samples. To address these limitations, future research should establish a more complete causal framework employing Noggin conditional overexpression transgenic mice and recombinant BMP4 protein interventions. Moreover, longitudinal monitoring of Noggin levels in serum and airway lavage fluid from preterm infants within clinical prospective cohorts is necessary to assess its sensitivity and specificity as an early predictive biomarker for BPD.

## 5 Conclusion

This study suggests that Noggin may function as an important upstream regulator promoting senescence in pulmonary microvascular endothelial cells under hyperoxic conditions, thereby supporting the involvement of the Noggin–BMP4–p53/p21 signaling axis in vascular injury and impaired alveolar development in BPD. These findings enhance the current understanding of the vascular–epithelial coupling mechanisms underlying BPD and provide a potential framework for future studies exploring targeted therapeutic strategies aimed at modulating signaling pathway remodeling and cellular senescence.

## Data Availability

Publicly available datasets were analyzed in this study. This data can be found here: https://www.ncbi.nlm.nih.gov/geo/query/acc.cgi?acc=GSE32472. All other data generated in this study are included in the article and **Supplementary Material**. Raw experimental data are available from the corresponding author upon reasonable request.
