# Supplementary material for: Hyperoxia promotes bronchopulmonary dysplasia via Noggin-mediated BMP4 antagonism and cellular senescence
Source: Front Physiol. 2026 Apr 20;17:1761135. doi: 10.3389/fphys.2026.1761135 (PMC13136015; doi:10.3389/fphys.2026.1761135)
Supplement: Supplementary file 1 [file Supplementaryfile1.docx]

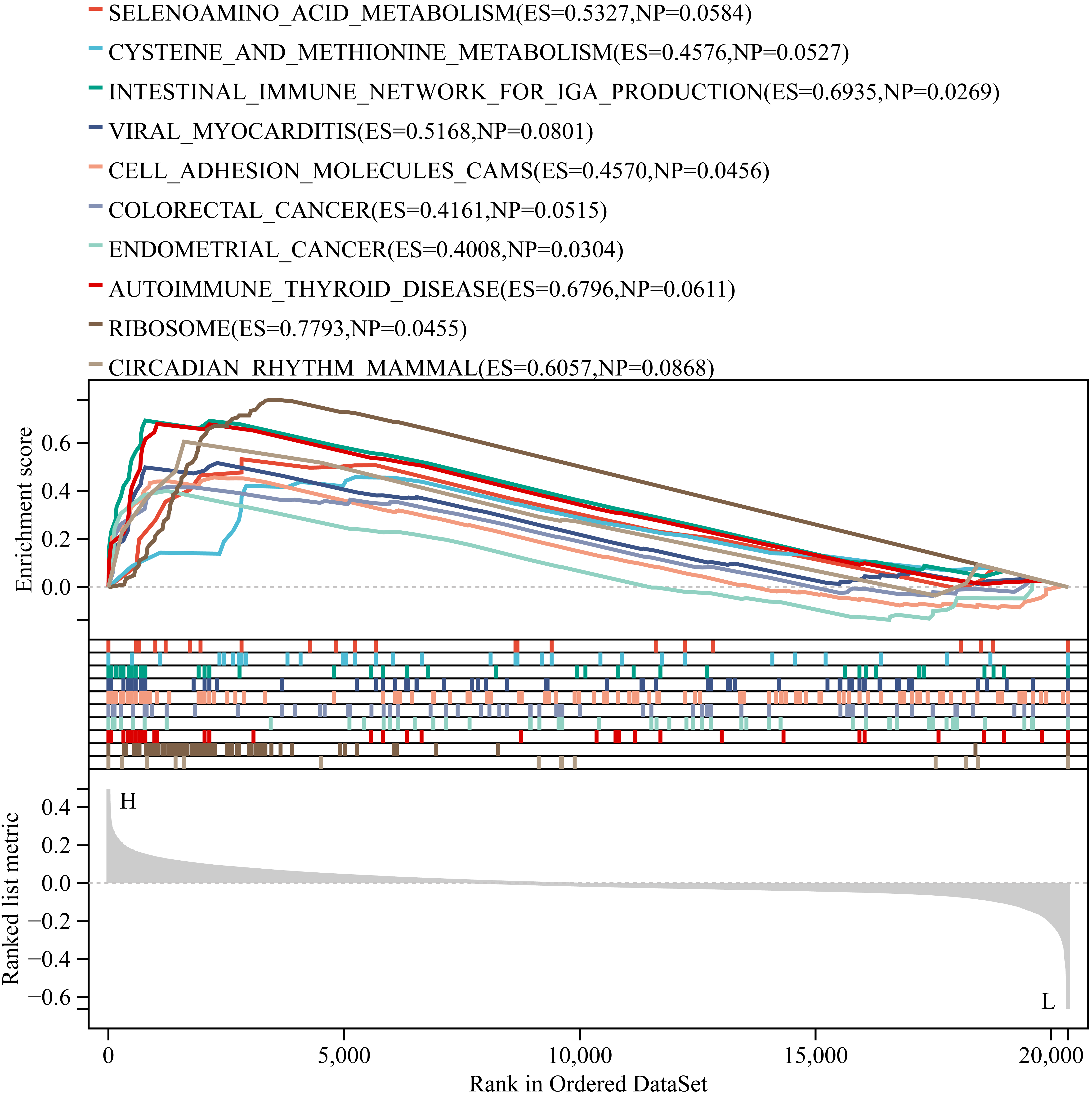


**Figure S1.** Gene Set Enrichment Analysis (GSEA). GSEA plot based on the KEGG database illustrating the top 10 significantly enriched pathways. The curves represent the running enrichment score (ES) for each gene set, and the vertical lines indicate the positions of individual pathway genes within the ranked list.


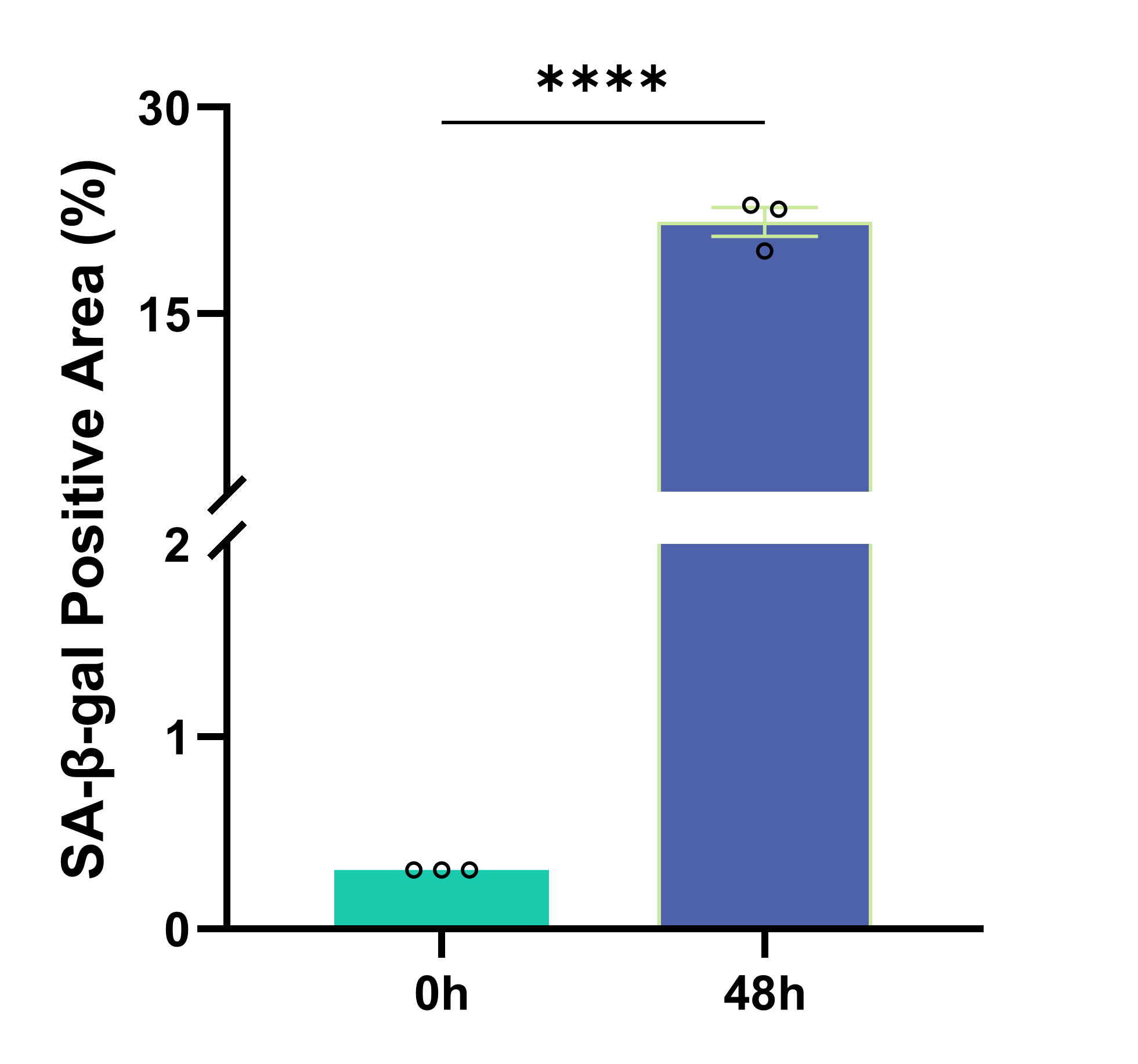


**Figure S2.** Quantification of SA-β-gal staining in HPMECs. The proportion of SA-β-gal positive area significantly increased in the hyperoxia-exposed group (48h) compared to normoxic controls (0h) (21.67% vs. 0.31%). Data are presented as mean ± SD (n = 3 independent fields/group). **** p < 0.0001.


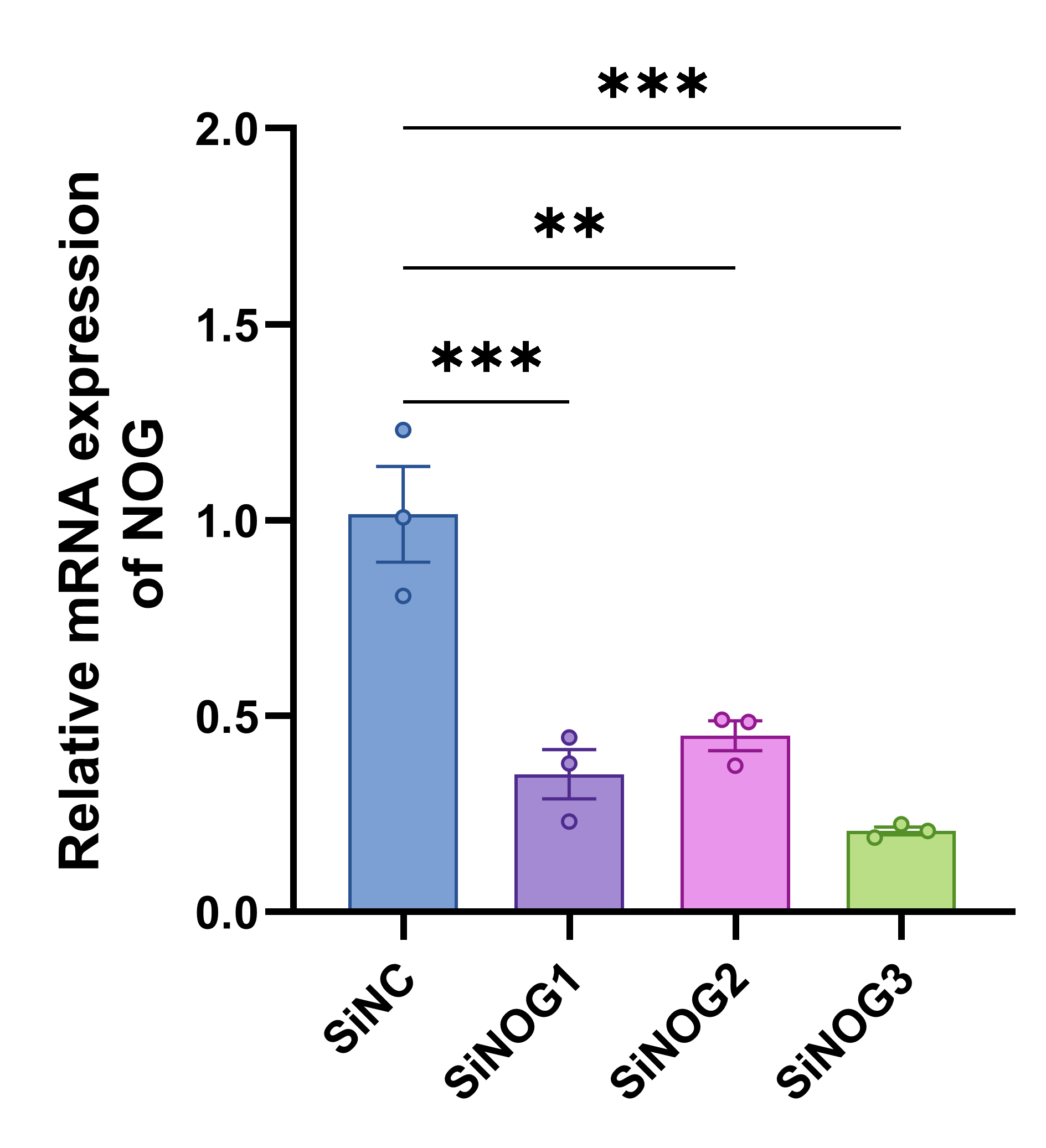


**Figure S3.** NOG knockdown efficiency. Relative NOG mRNA expression in HPMECs following transfection with negative control (SiNC) or NOG-specific siRNAs (SiNOG1-3). Data are presented as mean ± SD. ** P < 0.01, *** P < 0.001 vs. SiNC group.


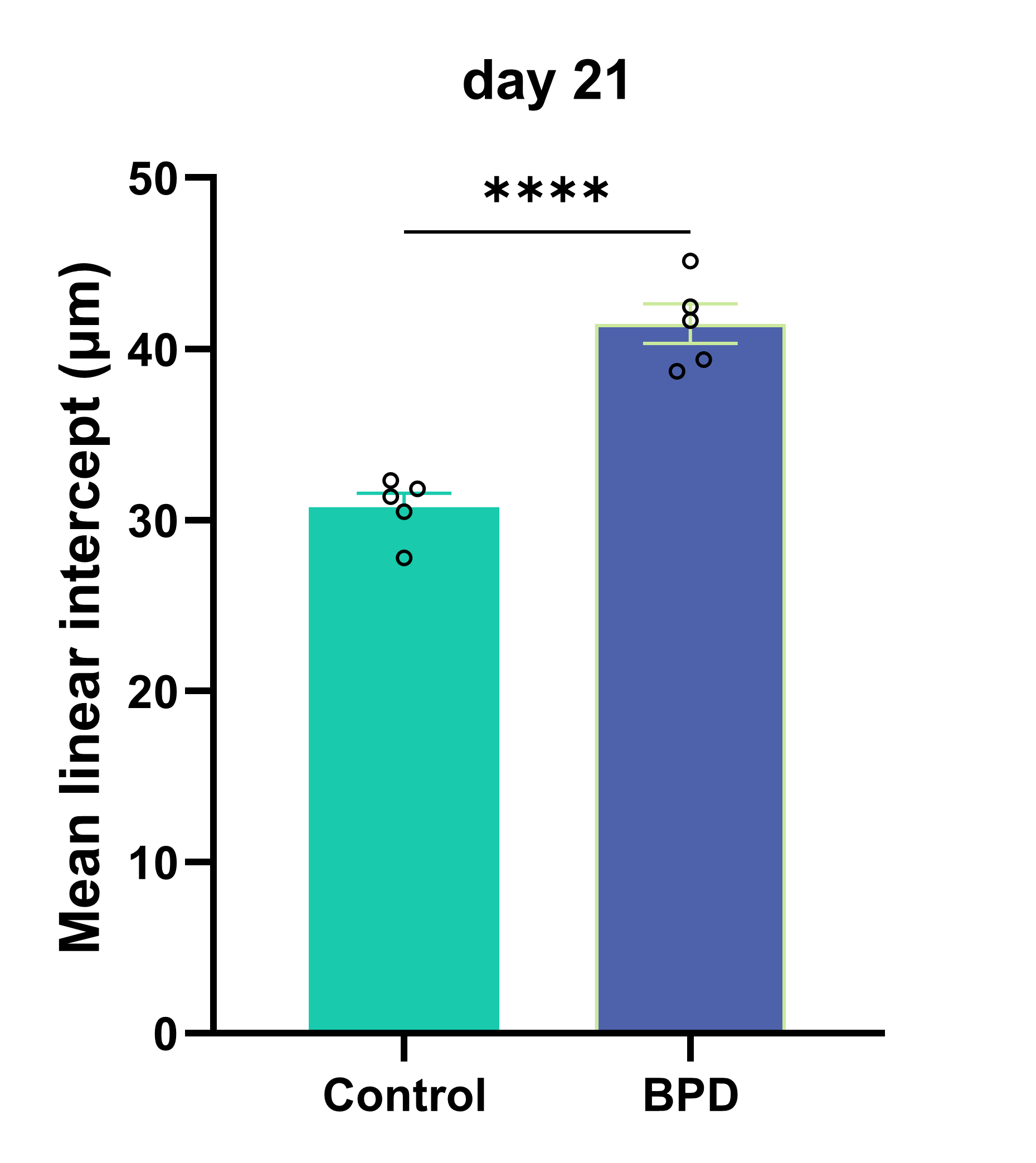


**Figure S4.** Quantitative morphometric analysis of alveolar simplification at postnatal day 21. The bar graph illustrates the MLI of lung tissues from the control and BPD groups at day 21. Data are presented as mean ± SD (n = 5 per group). ****P < 0.0001 vs. Control.
