# Supplementary material for: Hyperoxia promotes bronchopulmonary dysplasia via Noggin-mediated BMP4 antagonism and cellular senescence
Source: Front Physiol. 2026 Apr 20;17:1761135. doi: 10.3389/fphys.2026.1761135 (PMC13136015; doi:10.3389/fphys.2026.1761135)
Supplement: Supplementary file 2 [file Table1.docx]

# Table 1

# siRNA sequences for human NOG gene

| Gene | Sequence (5'→3') |
| --- | --- |
| SiNOG1 | F:GCCAGCACUAUCUCCACAUTT  R:AUGUGGAGAUAGUGCUGGCTT |
| SiNOG2 | F:GAGCGAGAUCAAAGGGCUATT  R:UAGCCCUUUGAUCUCGCUCTT |
| SiNOG3 | F:GGCAAGAAGCAGCGCCUAATT  R:UUAGGCGCUGCUUCUUGCCTT |
| NC | F:UUCUCCGAACGUGUCACGUTT  R:ACGUGACACGUUCGGAGAATT |
| NC-CY3 | F:UUCUCCGAACGUGUCACGUTT  R:ACGUGACACGUUCGGAGAATT-CY3 |
| SiGAPDH | F:GUGGAUAUUGUUGCCAUCATT  R:UGAUGGCAACAAUAUCCACTT |

SiNOG1/2/3: Noggin siRNA #1/2/3

NC: negative control

NC-CY3: Cy3 fluorescence-labeled negative control

SiGAPDH: siRNA GAPDH positive control

# Table 2

# Human gene primer sequences

| Gene | Sequence (5'→3') | bp |
| --- | --- | --- |
| NOG | F:GTACAAAGAGACGGGGGAAC  R:TTCGAGGTCCAAGGAAAACC | 91 |
| BMP4 | F:AGCTTCCACCACGAAGAACAT  R:AAGCCCCTTTCCCAATCAGG | 170 |
| P53 | F:TCCCCTGCCATTTTGGGTTT  R:GCAGGCCAACTTGTTCAGTG | 117 |
| P21 | F:GCTGCCGAAGTCAGTTCCT  R:GGTTCTGACGGACATCCCC | 112 |
| β-actin | F:AAACTGGAACGGTGAAGGTG  R:AGAGAAGTGGGGTGGCTTTT | 171 |

# Table 3

# Rat gene primer sequences

| Gene | Sequence (5'→3') | bp |
| --- | --- | --- |
| Nog | F:AGGAGGAAGTTACAGATGTGGC  R:GCACAGACTTGGATGGCTTAC | 172 |
| Bmp4 | F:GAAGAAGAGCAGAGCCAGGG  R:TGTTCTCCAGATGTTCTTCGTGA | 103 |
| Tp53 (p53) | F:CCCCTGAAGACTGGATAACTGT  R:ATTAGGTGACCCTGTCGCTG | 146 |
| Cdkn1a (p21) | F:CCTAAGCGTACCGTCCAGAG  R:GAGAGCAGCAGATCACCAGATTA | 141 |
| β-actin | F:CCCGCGAGTACAACCTTCTTG  R:CCCGCGAGTACAACCTTCTTG | 71 |
